# Haemostatic Gene Expression in Cancer-Related Immunothrombosis: Contribution for Venous Thromboembolism and Ovarian Tumour Behaviour

**DOI:** 10.3390/cancers16132356

**Published:** 2024-06-27

**Authors:** Valéria Tavares, Joana Savva-Bordalo, Mariana Rei, Joana Liz-Pimenta, Joana Assis, Deolinda Pereira, Rui Medeiros

**Affiliations:** 1 Molecular Oncology and Viral Pathology Group, Research Center of IPO Porto (CI-IPOP)/Pathology and Laboratory Medicine Dep., Clinical Pathology SV/RISE@CI-IPOP (Health Research Network), Portuguese Oncology Institute of Porto (IPO Porto)/Porto Comprehensive Cancer Centre (Porto. CCC), 4200-072 Porto, Portugal; valeria.tavares@ipoporto.min-saude.pt; 2 ICBAS—Instituto de Ciências Biomédicas Abel Salazar, Universidade do Porto, 4050-313 Porto, Portugal; 3 Faculty of Medicine of the University of Porto (FMUP), 4200-072 Porto, Portugal; jpimenta@chtmad.min-saude.pt; 4 Department of Medical Oncology, Portuguese Institute of Oncology of Porto (IPO Porto), 4200-072 Porto, Portugal; joana.sa@ipoporto.min-saude.pt (J.S.-B.); dpereira@ipoporto.min-saude.pt (D.P.); 5 Department of Gynaecology, Portuguese Institute of Oncology of Porto (IPO Porto), 4200-072 Porto, Portugal; marianacruzrei@gmail.com; 6 Department of Medical Oncology, Centro Hospitalar de Trás-os-Montes e Alto Douro (CHTMAD), 5000-508 Vila Real, Portugal; 7 Clinical Research Unit, Research Center of IPO Porto (CI-IPOP)/RISE@CI-IPOP (Health Research Network), Portuguese Oncology Institute of Porto (IPO Porto)/Porto Comprehensive Cancer Center (Porto. CCC), 4200-072 Porto, Portugal; joana.assis@ipoporto.min-saude.pt; 8 Faculty of Health Sciences, Fernando Pessoa University, 4200-150 Porto, Portugal; 9 Research Department, Portuguese League Against Cancer (NRNorte), 4200-172 Porto, Portugal

**Keywords:** ovarian neoplasms, prognosis, thrombosis, inflammation, genes, liquid biopsy

## Abstract

**Simple Summary:**

The exploration of prognostic biomarkers in ovarian cancer (OC) persists due to the daunting challenge of therapy resistance and poor patient outcomes. Dysregulated components of haemostasis are recognized as pivotal players in OC pathogenesis, contributing to tumour growth, metastasis, and the onset of cancer-associated thrombosis (CAT). Drawing upon the concept of immunothrombosis, which elucidates the intricate crosstalk among immune cells, platelets, and endothelial cells, this study investigated the expression of haemostasis-related genes in peripheral blood entities (particularly platelets and immune cells). The aim was to uncover prognostic markers, potential therapeutic targets for cancer management, and CAT predictors. Lower pre-chemotherapy *F3* and *F8* expression levels were significantly associated with increased CAT susceptibility post tumour diagnosis. The latter was also associated with shorter progression-free and overall survival. These findings point out the prognostic potential of these genes in OC in the context of immunothrombosis. Validation in larger cohorts is essential for clinical translation.

**Abstract:**

Ovarian cancer (OC) is the deadliest gynaecological malignancy. Identifying new prognostic biomarkers is an important research field. Haemostatic components together with leukocytes can drive cancer progression while increasing the susceptibility to venous thromboembolism (VTE) through immunothrombosis. Unravelling the underlying complex interactions offers the prospect of uncovering relevant OC prognostic biomarkers, predictors of cancer-associated thrombosis (CAT), and even potential targets for cancer therapy. Thus, this study evaluated the expression of *F3*, *F5*, *F8*, *F13A1*, *TFPI1*, and *THBD* in peripheral blood cells (PBCs) of 52 OC patients. Those with VTE after tumour diagnosis had a worse overall survival (OS) compared to their counterparts (mean OS of 13.8 ± 4.1 months and 47.9 ± 5.7 months, respectively; log-rank test, *p* = 0.001). Low pre-chemotherapy *F3* and *F8* expression levels were associated with a higher susceptibility for OC-related VTE after tumour diagnosis (χ^2^, *p* < 0.05). Regardless of thrombogenesis, patients with low baseline *F8* expression had a shorter progression-free survival (PFS) than their counterparts (adjusted hazard ratio (aHR) = 2.54; *p* = 0.021). Among those who were not under platelet anti-aggregation therapy, low *F8* levels were also associated with a shorter OS (aHR = 6.16; *p* = 0.006). Moving forward, efforts should focus on external validation in larger cohorts.

## 1. Introduction

Worldwide, ovarian cancer (OC) stands as the eighth most common and deadliest cancer among women, with approximately 324,000 new cases and 206,000 reported deaths in 2022 [1]. The disease is regarded as the most lethal gynaecological malignancy, with a 5-year survival rate lower than 50% in most countries [2]. Over the last few years, significant advancements have been made in OC treatment, with the development of new therapeutic approaches. However, given the disease heterogeneity, more and better predictive and/or prognostic biomarkers are needed to personalise disease management, reduce side effects, and prolong patient survival [3].

Like other solid tumours, OC is known to modulate the activity of platelets, endothelial cells, and leukocytes at the tumour microenvironment (TME) to fuel tumourigenesis [4]. Under physiological conditions, these components have an active role in haemostasis, driving continuous blood circulation and preventing coagulopathies, while gatekeeping vascular integrity. However, up to 50% of all cancer patients and 90% of those with metastases exhibit haemostatic abnormalities, including haemorrhage and thrombosis events [5]. Cancer-associated thrombosis (CAT), comprising both arterial and venous events, is a common paraneoplastic syndrome and a major cause of morbimortality [6,7]. Although arterial thrombosis may occur, most CAT events involve the veins [8]. Patients with OC have an incidence of venous thromboembolism (VTE) events, including deep venous thrombosis (DVT) and pulmonary embolism (PE), ranging from 10% to 30%, placing them among the cancer populations most affected by VTE [9]. This is critical given that VTE stands as the second leading cause of mortality among oncological patients [10].

Leukocytes (particularly monocytes and neutrophils), platelets, and endothelial cells are known to establish a complex interplay within TME, leading to a process known as immunothrombosis or thromboinflammation. This novel concept links inflammation and thrombogenesis to cancer progression [11,12]. In settings of inflammation (e.g., infection and cancer), leukocytes express numerous haemostatic proteins, including platelet activators, procoagulant factors, anticoagulant proteins, and fibrinolytic modulators [13]. Thus, the gene expression profile of these cells in OC patients may inform their thrombotic potential, helping anticipate VTE events. Importantly, given the contribution of haemostatic components in tumour growth and dissemination, this could lead to the identification of prognostic biomarkers of OC regardless of VTE, as well as potential therapeutic targets for cancer management. This is relevant considering the increasing recognition of liquid biopsies as non-invasive tests for assessing cancer patient prognosis and monitoring [14]. Hence, this study evaluated the expression of haemostasis-related genes in peripheral blood cells (PBCs) of OC patients, exploring their impact on OC-related VTE development and patients’ clinical outcomes.

## 2. Materials and Methods

### 2.1. Patient Enrolment

Adult Caucasian patients with confirmed ovarian carcinoma, who were admitted for frontline treatment at the Clinic of Gynaecology of the Portuguese Oncology Institute of Porto (IPO Porto) between March 2017 and August 2022, were enrolled in a retrospective cohort study. The standard treatment protocol consisted of cytoreductive surgery and chemotherapy using platinum-based agents (cisplatin or carboplatin), typically combined with taxanes (paclitaxel or docetaxel), administered every 21 days. The treatment approach (neoadjuvant, adjuvant, or chemotherapy alone for non-surgical candidates) determined the number of chemotherapy cycles, tailored to individual patient requirements and therapeutic responses. Exclusion criteria encompassed patients that (1) had synchronous and metachronous tumours, (2) were immunosuppressed and/or had autoimmune diseases, (3) had an acute infection at the time of cancer diagnosis, (4) were pregnant or in postpartum (here defined as lasting six weeks following childbirth) at OC diagnosis, (5) were receiving anticoagulation therapy because of other conditions rather than VTE, (6) had prothrombin G20210A (*F2* rs1799963) or factor V Leiden (*F5* rs6025) polymorphisms, and (7) did not provide informed consent. Applying these criteria, 52 OC patients were recruited, for whom biological material derived from peripheral blood samples before first-line chemotherapy was available in our biobank.

The demographical and clinical data of the patients were revised using their electronic medical records. Data on baseline full blood count and coagulation tests, namely prothrombin time (PT), international normalised ratio (INR), and activated partial thromboplastin (aPTT), were also retrieved. The most validated CAT risk assessment model (RAM)—the Khorana score (KS)—was determined for all patients with available data for the following parameters: the cancer site, baseline haemoglobin levels, leukocyte and platelet count, and body mass index (BMI). A cut-off of 2 was considered [8]. Cancer-associated VTE deemed an event taking place six months before to two years after OC diagnosis [15]. Active screening was not performed since it is not integrated into the standard clinical procedures at IPO Porto. The median follow-up period was 26.5 months, with a range of 1.0 to 82.0 months.

Each patient signed a written informed consent following the principles of the Helsinki Declaration. The study protocol has received approval from the ethics committee at the research centre of IPO Porto (CI-IPOP) (CES. 69/021).

### 2.2. Blood Sample Collection and Processing

Before and after first-line chemotherapy, peripheral blood samples were collected in EDTA-coated tubes using a standard venipuncture technique. Erythrocytes in the samples were lysed using a 1 × ammonium–chloride–potassium (ACK) solution. Subsequently, the samples were frozen at −20 °C for 20 min and then centrifugated for 10 min at 2500 rpm at room temperature. After removing the supernatant, the samples were sequentially washed with 1 × ACK solution and 1 × phosphate-buffered saline (PBS) solution. Each wash was followed by rounds of centrifugation for 10 min at 2500 rpm and room temperature to separate and remove the supernatant. The resulting pellet of PBCs was diluted with TriPure^®^ Isolation Reagent (Roche Applied Science, Penzberg, Germany) and conserved at −80 °C until use.

### 2.3. Gene Selection

One of the most studied mechanisms underlying CAT among OC patients is the tumour overexpression of the coagulation factor III (FIII), also known as tissue factor (TF). This coagulation factor triggers the extrinsic coagulation pathway, which along with the intrinsic pathway, converges into the common pathway leading to fibrin deposition [16]. Thus, haemostatic genes to be evaluated in this study were selected focusing on those related to coagulation factors and anticoagulants of the extrinsic and common coagulation pathways that (1) are expressed by platelets (here defined as messenger RNA (mRNA) presence) and leukocytes (Table 1), (2) are associated with VTE, (3) were previously implicated in tumour progression, and (4) had available TaqMan^®^ gene expression assays. Based on these criteria, *coagulation factor 3* (*F3*), *coagulation factor 5* (*F5*), *coagulation factor 8* (*F8*), *coagulation factor 13 A chain* (*F13A1*), *tissue factor pathway inhibitor* 1 (*TFPI1*), and *thrombomodulin* (*THBD*) were selected.

### 2.4. Total RNA Extraction

Total RNA from the peripheral blood fraction was isolated using the GRS RNA kit—Blood & cultured cells (#GK08.0100) (Grisp Research Resolutions^®^, Porto, Portugal). Importantly, the DNAse treatment (DNase I set (#GKC01.0100), Grisp Research Resolutions^®^, Porto, Portugal) duration was extended to guarantee the complete removal of genomic DNA, which would interfere with downstream applications. RNA purity and quantity were assessed using the NanoDrop Lite spectrophotometer (Thermo Fisher Scientific, Waltham, MA, USA). Post extraction, RNA samples were frozen at −80 °C until use.

### 2.5. cDNA Synthesis

The RNA samples (150 ng) were reverse-transcribed into complementary DNA (cDNA) using the High-Capacity cDNA Reverse Transcription Kit (Applied Biosystems^®^, Carlsbad, CA, USA) according to the manufacturer’s protocol. All the conversions were carried out in a Mycycler^TM^ Thermal cycler (Bio-Rad Laboratories, Hercules, CA, USA) under the following cycling conditions: 25 °C for 10 min, 37 °C for 120 min, and 85 °C for 5 min. Negative controls (lacking RNA) were integrated into all reactions to assess false positives.

### 2.6. Gene Relative Quantification

Quantitative real-time polymerase chain reaction (qRT-PCR) was employed to evaluate gene expression levels using a StepOnePlus^TM^ qPCR system (Applied Biosystems^®^, Foster City, CA, USA). Each PCR reaction utilised 5 µL of 2 × TaqMan™ Fast Advanced Master Mix (Applied Biosystems^®^, Foster City, CA, USA), 3.0 µL of RNA and DNA-free water, 0.5 µL of 20 × TaqMan^TM^ Gene Expression Assays for *F3* (Hs01076029_m1), *F5* (Hs00914120_m1), *F8* (Hs00252034_m1), *F13A1* (Hs01114178_m1), *TFPI1* (Hs00409206_m1, which quantify both *TFPI* isoforms), *THBD* (Hs00264920_s1), *GAPDH* (Hs03929097_g1), and *ACTB* (Hs99999903_m1) as well as 1.5 µL of the cDNA sample, in a total volume of 10 µL. *GAPDH* and *ACTB* were tested as endogenous controls (housekeeping genes) due to their reported expression stability in human blood cells [31,32,33]. The cycling conditions were as follows: 50 °C for 2 min, 95 °C for 10 min, 45 cycles of 15 s at 95 °C, and 60 °C for 60 s. The expression levels of each sample’s target and housekeeping genes were quantified on the same plate. Quantification was carried out in triplicate, with negative controls (without cDNA) incorporated in all reactions for quality control. Cycle threshold (Ct) values with a standard deviation (SD) greater than 0.5 were excluded. The Thermo Fisher Connect platform (Thermo Fisher Scientific, Waltham, MA, USA) was employed to set the same baseline and threshold values in all plates, ensuring uniform generation of Ct values for all the target and housekeeping genes in each sample.

### 2.7. Statistical Analysis

Statistical analysis and graphing were carried out using IBM SPSS Statistics for Windows version 29 (IBM Corp., Armonk, NY, USA) and GraphPad Prism version 9.0.0 (GraphPad Software Inc., La Jolla, CA, USA), respectively.

The normalised relative expression of the target genes was assessed using the Livak method. Among the endogenous controls, *GAPDH* showed a more stable expression (meaning with the lowest SD values) than *ACTB*. Thus, *GAPDH* was used to perform the normalisation of gene expression. The interquartile range (IQR) method was employed to pinpoint severe outliers, which were excluded. For each gene, four profiles of expression were created as follows: profile 1 (low versus vs. high expression using the median value of gene expression level as cut-off), profile 2 (low vs. intermediate vs. high expression using terciles), profile 3 (low vs. high, where low included the combination of the first and second tercile and the latter was the third tercile), and profile 4 (low vs. high, where the first tercile was classified as low and the remaining terciles as high expression). All these profiles were evaluated in analyses employing gene-normalised relative expression as a nominal variable.

Patients were regarded as VTE-free if they remained without VTE or died without presenting the condition during a two-year follow-up period. The live patients with less than two years of follow-up were excluded from the VTE analysis.

Data normality was assessed by employing the Kolmogorov–Smirnov test or the Shapiro–Wilk test depending on the cohort size (N > 50 and N ≤ 50, respectively). Depending on the distribution, Pearson’s correlation coefficient test (P) or Spearman’s rank correlation coefficient test was computed to assess the relationship between the genes’ expression before first-line chemotherapy. Results were deemed relevant if *p* < 0.05 and the coefficient ≥0.500. The coefficient of determination (R^2^) was also reported.

Associations of VTE development and pre-chemotherapy (baseline) gene expression levels with patients’ characteristics (Table 2) were evaluated using the Chi-Square test (χ^2^), excluding patients with VTE before OC diagnosis. In this analysis, subgroup evaluations according to primary treatment (surgery vs. chemotherapy) were also conducted.

Statistical differences in the baseline gene expression levels according to VTE status (without VTE vs. VTE before OC diagnosis vs. VTE after OC diagnosis) were analysed using the Kruskal–Wallis test or one-way ANOVA followed by Dunnett’s test for multiple comparisons, depending on data normality. In this analysis, those with VTE before and after OC diagnosis were compared with VTE-free patients, respectively. χ^2^ was additionally employed for confirmation. Depending on data distribution, the paired *t*-test or Wilcoxon’s matched-pairs signed-rank test was used to assess the impact of first-line chemotherapy on gene expression levels.

The study evaluated progression-free survival (PFS) and overall survival (OS) as clinical outcomes. PFS was defined as the time from the treatment initiation to the first occurrence of tumour progression or relapse, the patient’s death, or last clinical examination. The former was assessed following the Response Evaluation Criteria in Solid Tumours (RECIST) criteria version 1.1 (RECIST 1.1) [34]. Regarding OS, it was the period between cancer diagnosis and death related to all causes or the patient’s last follow-up date. Survival curves were generated using the Kaplan–Meier method, and survival probabilities were evaluated using the log-rank test. The influence of gene expression on the risk of OC progression and mortality was confirmed using the Cox regression model. A multivariate Cox analysis was performed for the relevant genes, adjusting for patient characteristics with prognostic value according to univariate Cox analyses. In these analyses, patients with VTE before tumour diagnosis were excluded.

In all analyses, a *p*-value lower than 0.05 was considered statistically significant. Furthermore, a *p*-value ranging between 0.05 and 0.06 was deemed marginally significant.

## 3. Results

### 3.1. Patients Characteristics

Demographic and clinical factors of the OC patients are presented in Table 2.

**Table 2:** Characteristics of ovarian cancer (OC) patients (N = 52).

Variable	n (%)
Age at OC diagnosis (years) *	63.6 ± 12.0
≥64	27 (51.9)
**Hormonal status at OC diagnosis**	
Postmenopausal	40 (76.9)
Baseline BMI (kg/m^2^) *	26.8 ± 4.9
≥27.0	21 (40.4)
**ECOG PS at OC diagnosis**	
>1	7 (13.5)
Baseline haemoglobin levels (U/mL) *	12.4 ± 1.4
<12.4	25 (48.1)
Baseline platelet count (×10^9^/L) **	296.0 [164.0; 572.0]
≥296.0	25 (48.1)
Baseline leucocyte count (×10^9^/L) *	7.9 ± 2.3
≥7.9	25 (48.1)
Baseline neutrophil count (×10^9^/L) *	5.1 ± 2.1
≥5.1	25 (48.1)
Baseline monocyte count (×10^9^/L) **	0.6 [0.3; 1.4]
≥0.6	24 (46.2)
Baseline lymphocyte count (×10^9^/L) **	1.5 [0.6; 4.3]
≥1.5	24 (46.2)
Baseline PT (s) **	14.2 [11.4; 31.2]
≥14.2	22 (42.3)
Baseline INR **	1.1 [1.0; 2.2]
≥1.1	21 (40.4)
Baseline aPTT (s) *	27.1 ± 2.4
≥27.1	21 (40.4)
**KS**	
≥2	22 (42.3)
**Platelet anti-aggregation therapy at OC diagnosis**	8 (15.4)
Anticoagulation therapy at OC diagnosis ***	3 (5.8)
**OC-related inherited mutations**	5 (9.6)
**Tumour histology**	
Serous	44 (84.6)
Clear cell	3 (5.8)
Endometroid	1 (1.9)
Mixed	2 (3.8)
Unusual	2 (3.8)
**Histological grade**	
High	49 (94.2)
**FIGO stage #**	
I/II	10 (19.2)
III/IV	42 (80.8)
Baseline CA-125 levels (U/mL) **	1067.0 [7.7; 10,184.0]
≥1067	26 (50.0)
**Upfront treatment**	
Surgery and adjuvant chemotherapy	23 (44.2)
Neoadjuvant chemotherapy and surgery	2 (3.8)
Neoadjuvant chemotherapy, surgery and adjuvant chemotherapy	14 (26.9)
Chemotherapy only	13 (25.0)
**Platinum sensitivity δ**	40 (76.9)
**Maintenance therapy**	
PARPi	16 (30.8)
bevacizumab	6 (11.5)

Baseline values were defined as those at tumour diagnosis, preceding treatment. * Presented as mean ± standard deviation since the variable had a normal distribution (Kolmogorov–Smirnov, *p* > 0.05). ** The variable was presented as median [minimum; maximum] due to its non-normally distributed nature. *** Patients with OC-related venous thromboembolism before OC diagnosis. # According to FIGO Cancer Report 2021 [35]. δ Those with disease progression six months after the completion of first-line platinum-based chemotherapy were deemed platinum-sensitive [3]. Some patients had missing information: 10 for aPTT, PT, and INR, five for monocyte and lymphocyte counts, four for KS, three for haemoglobin levels, platelet, leukocyte, and neutrophil counts, two for histological grade, and one for BMI, OC-related inherited mutations, and CA-125 levels. Abbreviations: aPTT, activated partial thromboplastin; BMI, body mass index; CA-125, cancer antigen 125; ECOG PS; Eastern Cooperative Oncology Group Performance Status; FIGO, International Federation of Gynecology and Obstetrics; INR, international normalised ratio; KS, Khorana score; OC, ovarian cancer; PARPi, Poly (ADP-ribose) polymerase inhibitors; PT, prothrombin time.

### 3.2. Impact of VTE on Patients’ Prognosis

Considering the patients with a two-year follow-up (N = 35), eight (22.9%) presented OC-related VTE, the majority being symptomatic (6 (75.0%)). Among the events, seven were DVT and one was PE. Those with thrombotic events before OC diagnosis (N = 3) present a mean period between VTE and tumour diagnosis of 2.3 ± 2.5 months. As for those with the condition after OC diagnosis (N = 5), the mean time to VTE occurrence was 4.6 ± 3.9 months.

Except for baseline leucocyte count, no significant association between VTE incidence and patients’ characteristics, including KS, was observed regardless of VTE timing (χ^2^, *p* > 0.05). Patients with venous thrombotic events more often presented a high baseline leucocyte count compared to their counterparts (7 vs. 1; χ^2^, *p* = 0.043).

**Figure 1:**
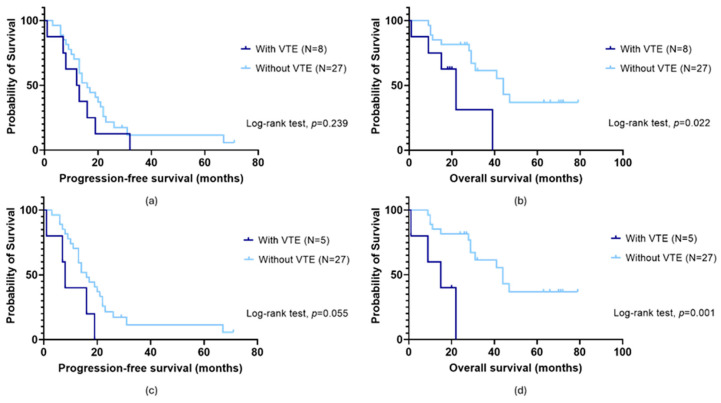
Progression-free survival (PFS) (**a**,**c**) and overall survival (OS) (**b**,**d**) by Kaplan–Meier and log-rank test for ovarian cancer (OC) patients according to venous thromboembolism (VTE) status. (**a**) No association between PFS and VTE (log-rank test, *p* = 0.239) was observed in the overall cohort (N = 35). (**c**) When patients with VTE before OC diagnosis were dismissed, a marginally significant impact was detected (long-rank test, *p* = 0.055). Specifically, those with OC-related VTE had a faster disease progression compared to their counterparts (mean PFS of 10.2 ± 3.2 months and 21.6 ± 3.8 months, respectively). (**b**) Considering the entire cohort (N = 35), a significant association between OS and VTE was observed (log-rank test, *p* = 0.022). Those with the condition had a lower survival time than their counterparts (mean OS of 22.2 ± 6.2 months and 47.9 ± 5.7 months, respectively). (**d**) The same was observed excluding those with VTE before OC diagnosis (log-rank test, *p* = 0.001). Specifically, patients with OC-related VTE and those without had a mean OS of 13.8 ± 4.1 months and 47.9 ± 5.7 months, respectively.

No significant impact of VTE on patients’ PFS was observed (log-rank test, *p* = 0.239; Figure 1a). In opposition, those with OC-related VTE demonstrated a lower survival time than their counterparts (mean OS of 22.2 ± 6.2 months and 47.9 ± 5.7 months, respectively; log-rank test, *p* = 0.022; Figure 1b). Excluding those with the condition before OC diagnosis, a marginally significant association was observed between VTE and patients’ PFS (long-rank test, *p* = 0.055; Figure 1c). Specifically, those with OC-related VTE had a faster disease progression compared to their counterparts (mean PFS of 10.2 ± 3.2 months and 21.6 ± 3.8 months, respectively). In the same subgroup, a negative impact of VTE on OS was also observed (mean OS of 13.8 ± 4.1 months and 47.9 ± 5.7 months, respectively; log-rank test, *p* = 0.001; Figure 1d).

### 3.3. Correlation between Baseline Gene Expression

**Figure 2:**
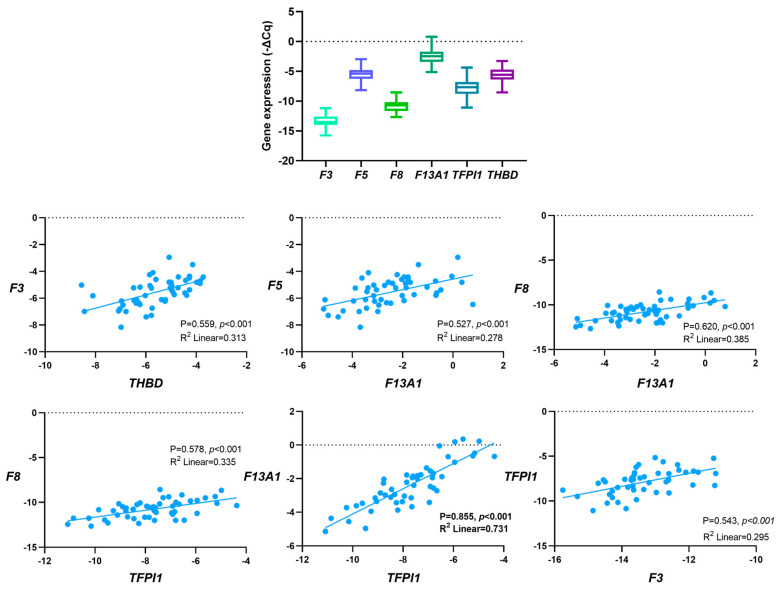
Correlation between baseline haemostatic genes’ expression in peripheral blood cells (PBCs) in a cohort of ovarian cancer (OC) patients (N = 52) by Pearson’s correlation coefficient (P) test.

Given the normal distribution (Kolmogorov–Smirnov, *p* > 0.05), Pearson’s correlation coefficient (P) test was employed to evaluate the relationship between the genes’ expression levels before first-line chemotherapy (Figure 2). Considering the entire cohort, positive correlations were detected between genes encoding for coagulation factors (*F5* and *F13A1*; *F8* and *F13A1*) and genes encoding for anticoagulants and coagulation factors (*F3* and *THBD*, *F8* and *TFPI1*, *F13A1* and *TFPI1* and *F3* and *TFPI1*). The strongest correlation was detected between *F13A1* and *TFPI1* expression (P = 0.855, *p* < 0.001, R^2^ = 0.731). All the correlations were also observed when excluding those with VTE before OC diagnosis.

### 3.4. Baseline Gene Expression and Patients’ Characteristics

For *F5* and *F13A1*, no significant association with patients’ characteristics was detected (χ^2^, *p* > 0.05), regardless of the expression profile and primary treatment (surgery vs. chemotherapy).

High pre-chemotherapy *F3* levels were more common among those with a high PT (≥14.2 s; profile 1, χ^2^, *p* = 0.032; profile 2, χ^2^, *p* = 0.040; profile 4, χ^2^, *p* = 0.040). In the subgroup analysis, according to primary treatment (surgery vs. chemotherapy), this association was confirmed among those first treated with surgery (≥14.2 s; profile 2, χ^2^, *p* = 0.006; profile 4, χ^2^, *p* = 0.024). In the same subgroup, high pre-chemotherapy levels of the gene were predominant among those at FIGO I/II stages compared to their counterparts (profile 2, χ^2^, *p* = 0.016; profile 3, χ^2^, *p* = 0.036).

High baseline *F8* levels were more common among patients with low INR (<1.1; profile 2, χ^2^, *p* = 0.004; profile 4, χ^2^, *p* = 0.031). Furthermore, its high levels were also more prevalent in the surgery group (profile 2, χ^2^, *p* = 0.042; profile 4, χ^2^, *p* = 0.039). In the chemotherapy subset (patients who were treatment-naïve upon sample collection), high pre-chemotherapy *F8* levels were predominant among younger patients (<64 years, profile 4, χ^2^, *p* = 0.043), those with high leucocyte count (≥7.9 × 10^9^/L; profile 3, χ^2^, *p* = 0.019), high platelet count (≥296.0 × 10^9^/L; profile 4, χ^2^, *p* = 0.025) and with a high KS (≥2; profile 4, χ^2^, *p* = 0.008) compared to their counterparts.

High pre-chemotherapy *TFPI1* levels were more common among patients with a high PT (≥14.2 s; profile 2, χ^2^, *p* = 0.026; profile 3, χ^2^, *p* = 0.019). In the chemotherapy group, high gene expression levels were predominant in patients with KS ≥ 2 (profile 1, χ^2^, *p* = 0.019).

Patients with elevated baseline *THBD* levels commonly presented a high KS (≥2; profile 2, χ^2^, *p* = 0.054; profile 3, χ^2^, *p* = 0.041). The subgroup analysis confirmed this association in the chemotherapy group (≥2; profile 2, χ^2^, *p* = 0.046; profile 3, χ^2^, *p* = 0.040).

### 3.5. Gene Expression and First-Line Chemotherapy

**Figure 3:**
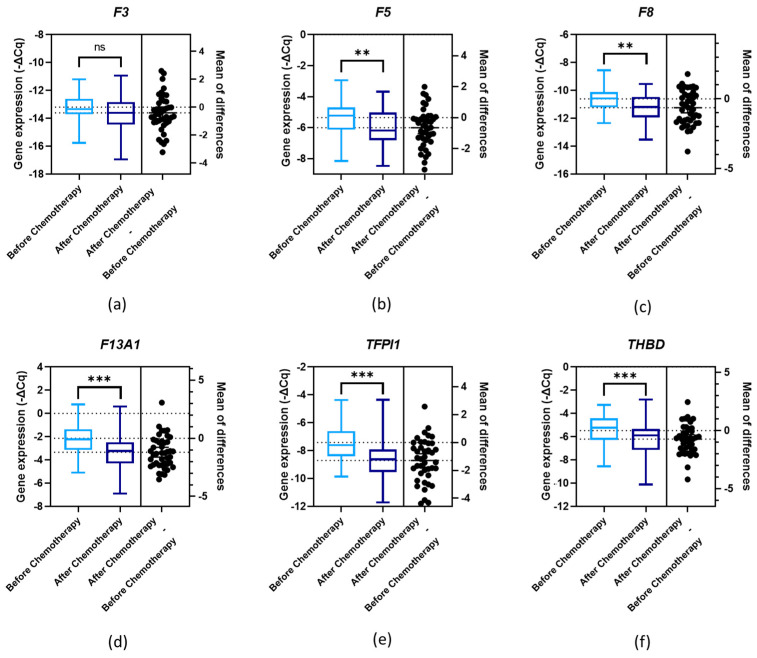
Normalised relative expression levels of the evaluated genes (−ΔCq) in peripheral blood cells (PBCs) in a cohort of ovarian cancer (OC) patients before and after first-line chemotherapy: (**a**) *F3* expression; (**b**) *F5* expression; (**c**) *F8* expression; (**d**) *F13A1* expression; (**e**) *TFPI1* expression; and (**f**) *THBD* expression; paired *t*-test, ** *p* < 0.01, *** *p* < 0.001; ns, non-significant.

Gene expression levels were evaluated according to first-line chemotherapy. Significant differences were observed (paired *t*-test, *p* < 0.05) between the genes’ expression levels before and after chemotherapy for *F5*, *F8*, *F13A1*, *TFPI1* and *THBD* (Figure 3b (*p* = 0.001), Figure 3c (*p* = 0.001), Figure 3d (*p* < 0.001), Figure 3e (*p* < 0.001) and Figure 3f (*p* < 0.001), respectively). As for *F3*, the difference was only marginally significant (paired *t*-test, *p* = 0.056; Figure 3a). In the subgroup analysis, according to primary treatment, except for *F3* (paired *t*-test, *p* = 0.445) and *F5* (paired *t*-test, *p* = 0.100), the expression levels of all genes significantly diminished after chemotherapy among those treated first with surgery. Regarding the chemotherapy group, only *F8* did not present a significant difference in expression levels after the treatment (paired *t*-test, *p* = 0.189).

### 3.6. Baseline Gene Expression and OC-Related VTE

**Figure 4:**
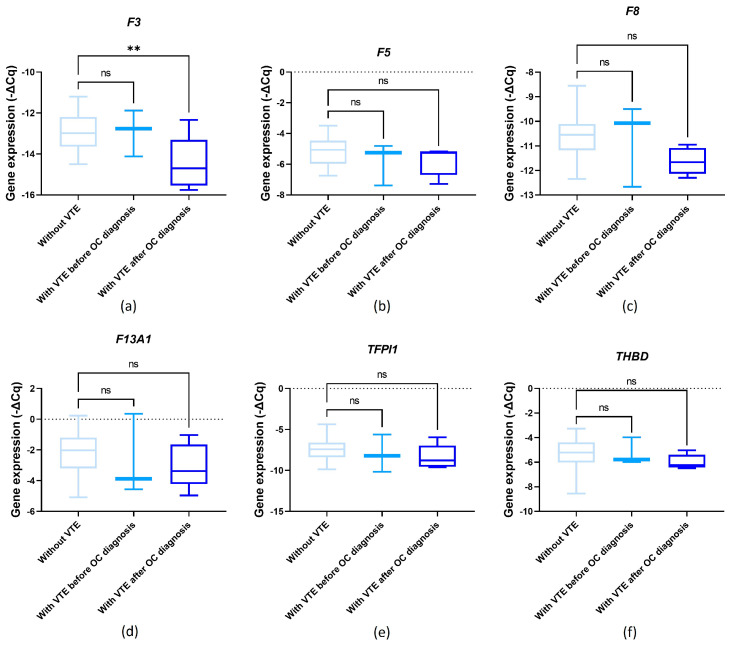
Normalised relative expression levels of the evaluated genes (−ΔCq) in peripheral blood cells (PBCs) in a cohort of ovarian cancer (OC) patients before first-line chemotherapy and in the context of venous thromboembolism (VTE): (**a**) *F3* expression; (**b**) *F5* expression; (**c**) *F8* expression; (**d**) *F13A1* expression; (**e**) *TFPI1* expression; and (**f**) *THBD* expression; one-way ANOVA followed by Dunnett’s test for multiple comparisons, ** *p* < 0.01; ns, non-significant.

The influence of pre-chemotherapy genes’ expression levels on OC-related VTE incidence was assessed. No significant differences were detected for *F5*, *F13A1*, *TFPI1* and *THBD* comparing VTE and VTE-free patients (Figure 4b,d–f; one-way ANOVA followed by Dunnett’s test, *p* > 0.05). Regarding *F3*, its pre-chemotherapy expression levels were significantly decreased in patients who later presented OC-related VTE compared to those without venous thrombogenesis (one-way ANOVA followed by Dunnett’s test, *p* = 0.008; Figure 4a). This finding was confirmed by χ^2^ (profile 2, *p* = 0.028; profile 4, *p* = 0.016). A marginal association was detected for *F8*. Namely, its pre-chemotherapy expression levels were decreased among those who later had a diagnosis of OC-related VTE compared to those without CAT (one-way ANOVA followed by Dunnett’s test, *p* = 0.057; Figure 4c). This finding was also corroborated by χ^2^ (profile 1, *p* = 0.051; profile 2, *p* = 0.028).

### 3.7. Impact of Baseline Gene Expression on Patients’ Prognosis

For *F3*, *F5*, *F13A1*, *TFPI1* and *THBD*, the baseline expression of these haemostatic genes had no significant impact on the patient’s clinical outcomes, regardless of the expression profile (log-rank test and univariable and multivariable Cox analyses; *p* > 0.05).

**Figure 5:**
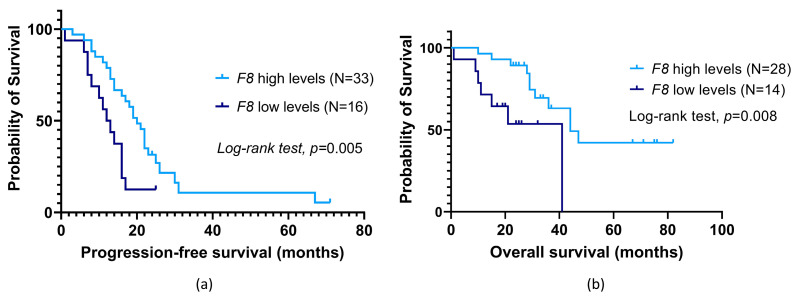
Progression-free survival (PFS) (**a**) and overall survival (OS) (**b**) by Kaplan–Meier and log-rank test for ovarian cancer (OC) patients according to *F8* baseline expression in peripheral blood cells (PBCs). (**a**) Patients with low expression levels had a lower PFS than their counterparts (profile 4; mean PFS of 12.8 ± 1.6 months and 23.7 ± 3.5 months, respectively; log-rank test, *p* = 0.005). (**b**) Dismissing patients under platelet anti-aggregation therapy at OC diagnosis, those with low expression had an inferior OS compared to their counterparts (profile 4; mean OS of 27.5 ± 4.5 months and 53.7 ± 5.9 months, respectively; log-rank test, *p* = 0.008).

Regarding *F8*, its low baseline expression was found to be associated with a lower time to OC progression compared to their counterparts (profile 4; mean PFS of 12.8 ± 1.6 months and 23.7 ± 3.5 months, respectively, log-rank test, *p* = 0.005; Figure 5a). The patients with a low expression of this haemostatic gene had almost a three-fold increase in the risk of disease progression (profile 4, hazard ratio (HR) = 2.56, 95% confidence interval (95% CI), 1.28–5.15, *p* = 0.008). This association was observed regardless of primary treatment (profile 4, adjusted HR (aHR) = 2.23, 95% CI, 1.08–5.59, *p* = 0.030). The negative effect of low *F8* baseline expression was corroborated in a multivariable Cox analysis adjusted for patients’ age at OC diagnosis and presence of metastasis at disease diagnosis and/or during treatment (profile 4; aHR = 2.54, 95% CI, 1.15–5.58, *p* = 0.021; Table 3). As for patient survival, no significant association between baseline *F8* expression and patients’ OS was detected (log-rank test, *p* > 0.05). However, among those who were not under platelet anti-aggregation therapy at OC diagnosis, low pre-chemotherapy *F8* levels were associated with a decreased survival time compared to their counterparts (profile 4; mean OS of 27.5 ± 4.5 months and 53.7 ± 5.9 months, respectively; log-rank test, *p* = 0.008; Figure 5b). In this subgroup, patients with low gene expression had almost a four-fold increase in the risk of mortality (profile 4, HR = 3.80, 95% CI, 1.31–10.98, *p* = 0.014). This negative influence was also observed regardless of primary treatment (profile 4, aHR = 3.36, 95% CI, 1.09–10.29, *p* = 0.034). A multivariable Cox analysis adjusted for surgery and platinum sensitivity also corroborated the negative contribution of low *F8* baseline expression (profile 4, aHR = 6.16, 95% CI, 1.68–22.52, *p* = 0.006; Table 3).

## 4. Discussion

Haemostatic components in the OC microenvironment shape cancer growth and dissemination while heightening susceptibility to cancer-related VTE, which complicates patient management and negatively impacts prognosis [36]. Numerous mechanisms have been proposed for the hypercoagulable state seen in cancer patients [10]. Intriguingly, tumour cells can instigate a thrombo-inflammatory cascade by activating endothelial cells, platelets, and immune cells. Platelets and endothelial cells recruit neutrophils to the TME, while concurrently neutrophils activate platelets by generating neutrophil extracellular traps (NETs) and releasing pro-thrombotic mediators [4,37,38]. Moreover, platelets and endothelial cells interact with activated monocytes, contributing to immunosuppressive macrophage polarization. These immune cells also play a role in thrombus formation by expressing and releasing pro-thrombotic mediators, including TF [4,39].

The liver and vascular endothelium are deemed the primary sites for the synthesis of most coagulation factors and anticoagulant proteins (except for TF, FIV (calcium) and FVIII) [40]. However, platelets and immune cells can also produce these haemostatic proteins, especially in pathological states [18,20]. Indeed, platelets carry a pool of mRNA from megakaryocytes during thrombopoiesis, which could allow them to biosynthesize proteins [41]. Given the complex interplay between tumour cells, platelets, and immune and endothelial cells in the OC microenvironment, this study explored the implications of haemostatic gene expression patterns in PBCs among OC patients.

First, this study demonstrates a CAT incidence of 22.9%. Consistently, the literature reports an incidence of 10–30% for patients with this malignancy [9,42,43,44,45,46,47,48]. When focusing on those with the condition after OC diagnosis, a marginally significant association between VTE and patients’ PFS was detected (long-rank test, *p* = 0.055). Specifically, those with VTE presented a shorter PFS. Regardless of VTE timing, CAT patients exhibit a lower OS (log-rank test, *p* < 0.05). Regarding clinical characteristics with predictive impact on VTE, high leucocyte count was prevalent among VTE subjects (χ^2^, *p* = 0.043), supporting the involvement of these immune cells in OC-related VTE pathogenesis [49,50]. Notably, the pre-chemotherapy leucocyte count is one of the parameters of KS. This VTE RAM showed unfavourable performance in this study, as previously described [9]. These findings highlight the detrimental role of CAT in OC patients and underscore the need for better RAMs to improve primary thromboprophylaxis [51,52,53].

Correlations between the pro-clotting and anti-clotting gene expression were detected in this study, the strongest being between *F13A1* and *TFPI1* expression (P = 0.855, *p* < 0.001, R^2^ = 0.731). This suggests that PBCs might have a coordinated regulation of haemostatic genes to gatekeep the delicate balance of haemostasis. However, the precise dynamics of haemostatic gene regulation in these entities, mainly in pathological settings, represent an area of ongoing research.

The *F3* gene encodes for TF. Mainly expressed by extravascular tissue cells, TF is released into the bloodstream following vascular damage, binding to FVII [17]. The TF-activated FVII complex—the tenase complex—activates coagulation factor X (FX), which triggers thrombin generation [16]. Tumour overexpression of TF, a main promotor of OC-related VTE, is a common event and is associated with an unfavourable prognosis [16,54]. Beyond thrombosis, TF facilitates tumour growth and dissemination via numerous biological pathways, including the protease-activated receptor (PAR) 2 signalling [55,56]. Tumour cells can release TF, alone or within microvesicles, increasing the thrombogenic potential and aggravating cancer aggressiveness [54]. Furthermore, although with some debate, *F3* expression has been reported in platelets and immune cells, particularly monocytes, contributing to immunothrombosis in cancer and other pathological conditions [18,19,57,58].

In this study, high *F3* expression was more common among those with high PT (χ^2^, *p* < 0.05), suggesting a link to haemostatic abnormalities [59]. Likewise, patients at FIGO I/II stages presented higher *F3* levels (χ^2^, *p* < 0.05). Our research group’s previous studies suggest that certain VTE-related biomarkers may have a greater influence before metastasis, potentially facilitating this process [60]. Regarding the implications of first-line chemotherapy, a significant difference was only detected when focusing on those who underwent chemotherapy as the first therapy (paired *t*-test, *p* < 0.05). Multiple mechanisms can explain the difference in the expression. First, chemotherapy may have reduced the cancer-induced stimulation of PBCs by lowering the tumour burden. The therapeutic approach can also cause leukopenia, as well as negatively impact platelets and endothelial cells [61]. This could have influenced the interaction between these entities, decreasing *F3* expression in PBCs. However, further exploration is needed. Concerning thrombosis, lower baseline *F3* expression was associated with a higher risk for a later CAT event (one-way ANOVA followed by Dunnett’s test, *p* = 0.008). Although it seems counterintuitive, this association could be due to complex interactions within the haemostatic system or even intrinsic mechanisms underlying *F3* mRNA expression, processing, and storage in these cells, for which there are currently scarce data. Additionally, monocytes can release TF-bearing microvesicles that bind to activated platelets, endothelial cells, and neutrophils, which could also influence the kinetics of *F3* expression [62,63]. In-depth functional studies are required to dissect the dynamics of *F3* expression in PBCs. As for patient prognosis, no significant impact was observed.

The *F5* gene encodes for coagulation factor V (FV). When activated, this glycoprotein together with activated FX forms the prothrombinase complex [64]. This complex in the presence of a phospholipid surface and calcium (FIV) catalysed the conversion of prothrombin (coagulation factor II (FII) to thrombin (activated FII) in the common coagulation pathway [65]. Beyond its pro-clotting function, FV also inhibits coagulation in an intriguing process involving TFPI1 [66]. Regarding tumourigenesis, although not entirely comprehended, *F5* expression appears to have an oncogenic function [67,68,69]. In addition, FV generates thrombin, which has been associated with several tumourigenic processes [70]. Concerning its expression in PBCs, *F5* has been reported in leukocytes in pathological conditions [18,20,21]. As for platelets, it is unclear whether these cell fragments synthesise FV or the platelet-derived protein originated solely from megakaryocytes [20,71]. In this study, no association of *F5* levels with patients’ characteristics was detected (χ^2^, *p* < 0.05). Except for those who had surgery as the primary intervention, first-line chemotherapy significantly impacted *F5* expression levels (paired *t*-test, *p* < 0.05), which could be due to the previously described mechanisms. Lastly, no association of baseline *F5* levels with OC-related VTE development and clinical outcomes was observed (*p* > 0.05). Given the small cohort size, further studies are needed to gather more evidence on the role of this coagulation factor in PBCs among OC patients.

The *F8* gene encodes for FVIII. Once activated by thrombin, this glycoprotein accelerates the activation of FX in conjunction with activated coagulation factor IX (FIX), FIV and phospholipids [23,40]. In the bloodstream, FVIII is typically bound to the von Willebrand factor (vWF), which stabilises and protects it from premature proteolysis, while also facilitating its transportation to sites of endothelial damage [23]. The relationship between coagulation factors and VTE is thought to be largely attributed to deregulated FVIII and vWF levels, indicating a central role of this coagulation factor in venous thrombogenesis [72]. Although mainly synthesised in the liver, FVIII is also expressed by megakaryocytes/platelets and monocytes/macrophages as an additional strategy to restore haemostasis [18,20,22,23,24,25]. Several epidemiological studies have shown elevated FVIII circulating levels in cancer patients, with this expression having prognostic significance and being an independent predictor of VTE [73,74,75].

In the present study, high *F8* levels were more common among those with a lower INR, suggesting a potential link to haemostatic abnormalities (χ^2^, *p* < 0.05). Among those first treated with chemotherapy, high baseline *F8* levels were predominant among younger patients (profile 4, χ^2^, *p* = 0.043), those with high leucocyte count (profile 3, χ^2^, *p* = 0.019), high platelet count (profile 4, χ^2^, *p* = 0.025) and with a high KS (profile 4, χ^2^, *p* = 0.008). Concordantly, ageing has been shown to influence FVIII expression [76]. Concerning the association with leukocyte and platelet counts, it was expected given the reported expression profile in these entities [18,20,25,77]. As for the relationship between *F8* levels and KS, this finding also suggests a potential role in OC coagulome. Regarding the influence of first-line chemotherapy, the gene expression was significantly decreased after treatment (paired *t*-test, *p* < 0.001). However, no difference was detected when focusing on the patients who underwent chemotherapy as their first therapeutic intervention (paired *t*-test, *p* > 0.05). Concerning thrombosis, lower baseline *F8* expression was associated with a higher risk for a later OC-related VTE event (one-way ANOVA followed by Dunnett’s test, *p* = 0.057; χ^2^, profile 1, *p* = 0.051; profile 2, *p* = 0.028). Like *F3*, functional studies are required to dissect the dynamics of *F8* expression in PBCs as current knowledge remains limited, particularly in malignancy [78].

Considering clinical outcomes, patients with low baseline *F8* had almost a three-fold increase in the risk of disease progression (profile 4; aHR = 2.54, *p* = 0.021), supporting the finding that FVIII impacts tumourigenesis [73,74,78,79]. As for patient survival, only when focusing on patients who were not under platelet anti-aggregation therapy at OC diagnosis (in addition to excluding those receiving anticoagulation therapy upon tumour diagnosis) was a significant association between baseline *F8* expression and patients’ OS detected. Specifically, those with low gene expression had a six-fold increase in the risk of mortality (profile 4; aHR = 6.16, *p* = 0.006). Antiplatelet agents limit platelet aggregation and vasoconstriction, potentially reducing FVIII release from platelet alpha-granules [80]. Additionally, antiplatelet agents, such as acetylsalicylic acid (ASA), possess anti-inflammatory properties, which could affect leukocyte activity, potentially influencing their expression of *F8* [81]. To our knowledge, this is the first study linking *F8* expression in PBCs to OC patients’ prognosis. Overall, low baseline *F8* expression was associated with a high risk of CAT and an unfavourable prognosis regardless of thrombosis. This suggests that its expression by PBCs could be an attractive OC biomarker. Yet, more data are required.

Coagulation factor XIII (FXIII) is a transglutaminase that once activated crosslinks fibrin molecules to ensure blood clot stability [82]. In the plasma, FXIII circulates as a zymogen with two catalytic A subunits (FXIII-A_2_) and two non-catalytic B-subunits (FXIII-B_2_) [83,84]. The A subunit of FXIII (FXIIIA) is encoded by *F13A1*, which is expressed by cells of bone marrow and the mesenchymal lineage leading to three main pools of circulating FXIIIA: plasma, platelets and monocytes/macrophages [18,20,26]. In addition to coagulopathies, abnormal expression and/or activity of FXIII is proposed to influence cancer susceptibility and behaviour. Intriguingly, monocytes have been implicated in this mechanism [85,86]. In this study, no association between *F13A1* expression and patients’ characteristics was observed (χ^2^, *p* > 0.05). Concerning the influence of first-line chemotherapy, the intervention had a significant impact on the gene expression in all groups (paired *t*-test, *p* < 0.05). Contrariwise, no link to CAT and clinical outcomes was detected. Whether this finding is related to the small sample size or whether FXIIIA has a context-dependent role needs to be clarified.

The *TFPI1* (or *TFPI*) gene encodes for a protein with the same name (TFPI1/TFPI), known as the primary inhibitor of the extrinsic or TF coagulation pathway. There are two TFPI1 isoforms—TFPIα and TFPIβ. While the former is released by endothelial cells and activated thrombocytes, inhibiting both the prothrombinase and tenase complexes, the latter is presented at the endothelium surface and it is known to block the tenase complex more effectively [16]. In cancer settings, TFPI1 levels have been associated with VTE risk, metastatic potential, and all-cause mortality [87,88]. Indeed, this anticoagulant seems to act as a tumour suppressor [89]. In this study, first-line chemotherapy impacted *TFPI1* expression in all groups (paired *t*-test, *p* < 0.05). High *TFPI1* levels were more common among those with high PT and KS ≥ 2, the latter only among those that were first treated with chemotherapy (χ^2^, *p* < 0.05). Although these findings pinpoint a potential role in CAT, no significant contribution of *TFPI1* expression in PBCs was detected, neither in thrombosis nor in patients’ prognosis. More studies are needed to solidify these data.

The *THBD* gene encodes for thrombomodulin (TM), a thrombin receptor mainly expressed by endothelial cells, which reduces thrombin activity, preventing it from activating platelets and converting fibrinogen to fibrin [90]. Likewise, the TM-thrombin complex can terminate excessive coagulation by activating the protein C anticoagulation pathway, which further inactivates FV and FVIII [90,91]. This anticoagulant has been implicated in VTE in the broader population and cancer settings [92]. Overall, tumour expression of TM is linked to a favourable prognosis, decreasing the tumourigenic and metastatic potential, which can be explained by its anticoagulant and anti-inflammatory properties [93,94,95,96,97]. In this study, initial chemotherapy notably influenced *THBD* expression levels across all cohorts (paired *t*-test, *p* < 0.05). Those with elevated *THBD* levels commonly presented a high KS (χ^2^, *p* < 0.05), suggesting a potential role in the tumour coagulome. However, no association with OC-related VTE nor a prognostic significance of *THBD* expression in PBCs was observed, which requires further confirmation.

Overall, this research provides preliminary insights into the expression of haemostatic genes in PBCs and their potential implications for OC patients. While the study yielded promising results, it is essential to recognise its limitations. The main one was the small cohort size, which may have limited the statistical power. This was due to the relatively low incidence of OC and the necessity to control for major confounders associated with CAT and gene expression analysis in PBCs. Additionally, it would be important to assess gene expression in other settings, including healthy conditions and close to VTE occurrence in cancer and cancer-free individuals to study the expression dynamics in PBCs. An analysis close to thrombotic events was not possible given the retrospective nature of the study. Also, no active VTE screening was conducted, which could have led to the underestimation of asymptomatic events. Nevertheless, this study also has its strengths. Specifically, most of the major risk factors linked to VTE were accounted for. Furthermore, the findings could open avenues for liquid biopsies to predict OC-related VTE and assess patients’ prognosis.

## 5. Conclusions

Among gynaecological tumours, OC is deemed the most lethal. Due to active investigation, new therapeutic approaches have emerged to overcome OC resistance to treatment, paralleling the increased demand for disease biomarkers. Conversely, exploring the contributions of haemostasis deregulation to cancer progression is a potential avenue for identifying OC biomarkers and therapeutic targets. In the present research, the expression of haemostasis-related genes in PBCs was evaluated in a cohort of OC patients for their implications in CAT and patients’ prognosis (regardless of VTE). To our knowledge, this is the first study to do so. According to the findings, OC patients have a high tendency towards VTE, which negatively affects their prognosis. Furthermore, pre-chemotherapy *F3* and *F8* expression was found to predict OC-related VTE development. Although this should be analysed carefully given the small cohort size, these preliminary data point out the expression of haemostasis-related genes in platelets and leukocytes as a potential source of CAT predictors. The low expression of *F8* was also linked to shorter patients’ PFS and OS, supporting the two-way relationship between thrombosis and ovarian tumourigenesis. All in all, the expression of these genes in PBCs in a setting of cancer immunothrombosis could be an attractive tool to assess OC patient’s thromboembolic risk profile and prognosis non-invasively. This might pave the way for personalised thromboprophylaxis and better oncological treatment strategies to improve clinical outcomes. However, future studies should clarify the clinical applicability of these potential OC biomarkers in larger and more diverse cohorts. Also, it will be important to perform single-cell RNA sequencing to identify the specific cells responsible for the expression of these genes and their dynamics in physiological and pathological conditions.

## Figures and Tables

**Table 1:** Selected haemostasis-related genes and their expression profile.

Gene	Genomic Location	Main Source	Expression in Platelets and Immune Cells *
*F3*	1p21.3	Extravascular tissue cells (pericytes, fibroblasts, smooth and epithelial cells) [17]	Monocytes [18] Platelets [19]
*F5*	1q24.2	Hepatocytes [20]	Monocytes [18,20] Neutrophils [21] T cells [21] Platelets [20]
*F8*	Xq28	Liver sinusoidal endothelial cells [22] Hepatocytes [23] Megakaryocytes [24,25]	Monocytes [18] Macrophages [20] Platelets [25]
*F13A1*	6p25.1	Cells of bone marrow and the mesenchymal lineage [26]	Monocytes [18,20] Macrophages [20] Platelets [20,27]
*TFPI1*	2q32.1	Vascular endothelial cells [28] Platelets [18]	Monocytes [18] Macrophages [28]
*THBD*	20p11.21	Vascular endothelial cells [20]	Monocytes [18,20] Macrophages [20] Neutrophils [29] Dendritic cells [30]

* Defined as messenger RNA presence and protein synthesis.

**Table 3:** Multivariable Cox regression analysis on the risk of disease progression (N = 48) and risk of death (N = 41) among OC patients according to pre-chemotherapy *F8* levels (profile 4) in PBCs.

Variable	aHR	95% CI	*p*-Value	Event
*F8* baseline expression (Low vs. high ^1^)	2.54	**1.15–5.58**	**0.021**	**Risk of disease** **progression**
Age at OC diagnosis (≥64 vs. <64 years ^1^)	1.99	0.96–4.10	0.063	
Metastatic disease (Yes vs. no ^1^)	10.65	**2.46–46.12**	**0.002**	
*F8* baseline expression (Low vs. high ^1^)	6.16	**1.68–22.52**	**0.006**	**Risk of death**
Surgery (No vs. yes ^1^)	2.64	0.77–9.10	0.124	
Platinum sensitivity (Others vs. sensitive ^1^)	13.72	**3.35–56.22**	**<0.001**	

Bold values were deemed statistically significant. ^1^ Reference group. Abbreviations: aHR, adjusted hazard ratio; CI, confidence interval; OC, ovarian cancer; PBCs, peripheral blood cells.

## Data Availability

The data presented in this study are available on request from the corresponding author.
